# Aqua­trimeth­yl[2-(4-methyl­pyrimidin-2-ylsulfan­yl)acetato-κ*O*]tin(IV)

**DOI:** 10.1107/S1600536813012622

**Published:** 2013-05-15

**Authors:** Zhiqing Gao, Junhong Zhang, Xuyi Hao, Daqi Wang, Tingting Zhang

**Affiliations:** a Dongcheng College of Liaocheng University, Shandong 252059, People’s Republic of China; b College of Chemistry and Chemical Engineering, Liaocheng University, Liaocheng 252059, People’s Republic of China; c Tancheng Middle School, Linyin 276000, People’s Republic of China

## Abstract

In the title compound, [Sn(CH_3_)_3_(C_7_H_7_N_2_O_2_S)(H_2_O)], the Sn^IV^ atom has a distorted trigonal–bipyramidal coordination geometry, with one carboxyl­ate O atom of the 2-(4-methyl­pyrimidine-2-sulfan­yl)acetate ligand and the O atom of a water mol­ecule in axial positions, and three methyl groups in equatorial positions. In the crystal, mol­ecules are linked *via* O—H⋯O and O—H⋯N hydrogen bonds, forming double-stranded chains propagating along [010].

## Related literature

For related structures, see: Zhang *et al.* (2007 ▶); Zhu *et al.* (2011 ▶).
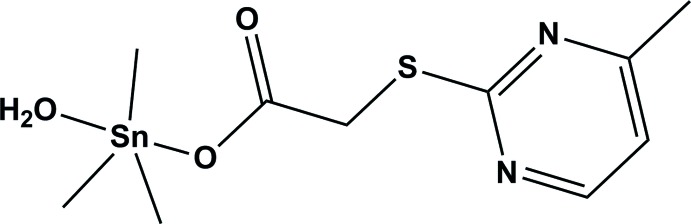


## Experimental

### 

#### Crystal data

[Sn(CH_3_)_3_(C_7_H_7_N_2_O_2_S)(H_2_O)]
*M* *_r_* = 365.01
Monoclinic, 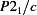

*a* = 7.7658 (4) Å
*b* = 11.0901 (4) Å
*c* = 18.8261 (8) Å
β = 113.575 (4)°
*V* = 1486.05 (11) Å^3^
*Z* = 4
Cu *K*α radiation
μ = 15.00 mm^−1^
*T* = 293 K
0.05 × 0.04 × 0.04 mm

#### Data collection

Xcalibur (Eos, Gemini) diffractometer
Absorption correction: multi-scan (*SADABS*; Bruker, 2007 ▶) *T*_min_ = 0.521, *T*_max_ = 0.585
8393 measured reflections
2666 independent reflections
2151 reflections with *I* > 2σ(*I*)
*R*_int_ = 0.059

#### Refinement

*R*[*F* ^2^ > 2σ(*F* ^2^)] = 0.047
*wR*(*F* ^2^) = 0.132
*S* = 1.05
2664 reflections
159 parameters
H-atom parameters constrained
Δρ_max_ = 1.52 e Å^−3^
Δρ_min_ = −0.78 e Å^−3^

### 

Data collection: *SMART* (Bruker, 2007 ▶); cell refinement: *SAINT* (Bruker, 2007 ▶); data reduction: *SAINT*; program(s) used to solve structure: *SHELXS97* (Sheldrick, 2008 ▶); program(s) used to refine structure: *SHELXL97* (Sheldrick, 2008 ▶); molecular graphics: *SHELXTL* (Sheldrick, 2008 ▶); software used to prepare material for publication: *SHELXTL*.

## Supplementary Material

Crystal structure: contains datablock(s) I, global. DOI: 10.1107/S1600536813012622/su2595sup1.cif

Structure factors: contains datablock(s) I. DOI: 10.1107/S1600536813012622/su2595Isup2.hkl

Additional supplementary materials:  crystallographic information; 3D view; checkCIF report

## Figures and Tables

**Table 1:** Hydrogen-bond geometry (Å, °)

*D*—H⋯*A*	*D*—H	H⋯*A*	*D*⋯*A*	*D*—H⋯*A*
O3—H3*D*⋯O1^i^	0.85	2.03	2.798 (6)	149
O3—H3*F*⋯N1^ii^	0.85	2.14	2.884 (7)	146

Symmetry codes: (i) 
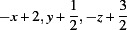
; (ii) 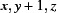.

**Fig. 1.**
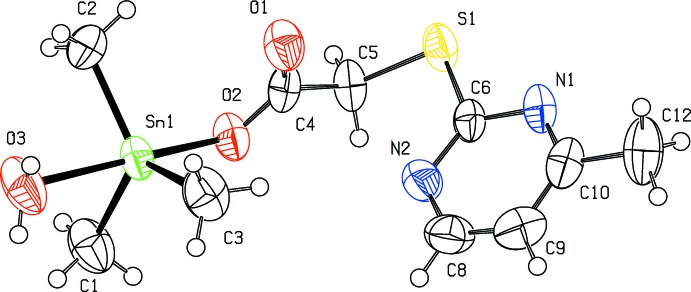
The molecular structure of the title complex, with atom labelling. Displacement ellipsoids are drawn at the 50% probability level.

**Fig. 2.**
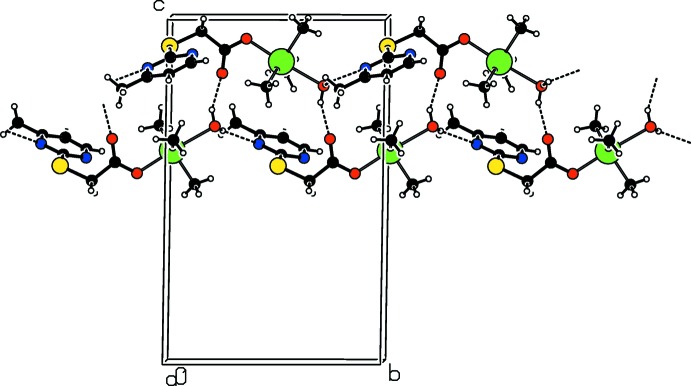
A partial view along the a axis of the crystal packing of the title compound. The hydrogen bonds are shown as dashed lines; see Table 1 for details.

## supplementary crystallographic information

### Comment

The title compound is a trimethyltin ester of 4-methylpyrimidine-2-thioglycolic acid, Fig. 1. The geometry of tin atom, Sn1, is a slightly distorted trigonal bipyramid, surrounded axially by atom O2 of the ligand and atom O3 of a water molecule. The axial angle O2—Sn1—O3 is 176.66 (16) Å close to a linear arrangement. Three carbon atoms of the methyl groups are located in the equatorially plane and the sum of the trigonal C—Sn—C angles is 358.1°, which illustrates that the three methyl C atoms and the tin atom are nearly coplanar. The Sn—O distances of 2.144 (4) Å and 2.488 (5) Å, are a little longer than the covalent bond length of tin and oxygen (Zhang *et al.*, 2007) but similar to the same distances reported for other triorganotin polymeric structures (Zhu *et al.*, 2011).

In the crystal, molecules are linked via O-H···O and O-H···N hydrogen bonds forming double-stranded chains propagating along [010]; see Table 1 and Fig. 2.

### Experimental

The reaction was carried out under an atmosphere of nitrogen. 4-methylpyrimidine-2-thioglycolic acid (1 mmol) and sodium ethanol (1 mmol) were added to a stirred solution of ethanol (30 ml) in a Schlenk flask and stirred for 30 min. Trimethyltin chloride (1 mmol) was then added to the reactor and the reaction mixture was stirred for 12 h at 353 K. The resulting clear solution was evaporated under vacuum. The product was crystallized from dichloromethane to yield colourless block-like crystals of the title compound [Yield 80%; M.p. 430 K]. Anal. Calcd (%) for C_10_H_18_N_2_O_3_SSn (Mr = 365.01): C, 32.90; H, 4.97; N, 7.67; Found (%): C, 32.87; H, 4.94; N, 7.71.

### Refinement

The water molecule H atoms were placed in calculated positions and treated as riding atoms: O-H = 0.85 Å with *U*_iso_(H) = 1.5*U*_eq_(O). The C-bound H atoms were also placed in calculated positions and treated as riding atoms: C—H = 0.93 and 0.96 Å, for CH and CH_3_ H atoms, respectively, with *U*_iso_(H) = 1.5*U*_eq_(C-methyl) and = 1.2*U*_eq_(C) for other H atoms. In the final cycles of refinement two reflections were omitted as (I_obs_-I_calc_)/ΣW > 10.

### Crystal data

**Table:**

[Sn(CH_3_)_3_(C_7_H_7_N_2_O_2_S)(H_2_O)]	*F*(000) = 728
*M**_r_* = 365.01	*D*_x_ = 1.631 Mg m^−^^3^
Monoclinic, *P*2_1_/*c*	Cu *K*α radiation, λ = 1.54184 Å
Hall symbol: -P 2ybc	Cell parameters from 2652 reflections
*a* = 7.7658 (4) Å	θ = 4.0–70.5°
*b* = 11.0901 (4) Å	µ = 15.00 mm^−^^1^
*c* = 18.8261 (8) Å	*T* = 293 K
β = 113.575 (4)°	Block, colourless
*V* = 1486.05 (11) Å^3^	0.05 × 0.04 × 0.04 mm
*Z* = 4	

### Data collection

**Table:**

Xcalibur (Eos, Gemini) [CHECK! BRUKER SOFTWARE?]diffractometer	2666 independent reflections
Radiation source: Enhance (Cu) X-ray Source	2151 reflections with *I* > 2σ(*I*)
Graphite monochromator	*R*_int_ = 0.059
Detector resolution: 16.2563 pixels mm^-1^	θ_max_ = 67.3°, θ_min_ = 4.7°
phi and ω scans	*h* = −6→9
Absorption correction: multi-scan (*SADABS*; Bruker, 2007)	*k* = −13→13
*T*_min_ = 0.521, *T*_max_ = 0.585	*l* = −22→21
8393 measured reflections	

### Refinement

**Table:**

Refinement on *F*^2^	Secondary atom site location: difference Fourier map
Least-squares matrix: full	Hydrogen site location: inferred from neighbouring sites
*R*[*F*^2^ > 2σ(*F*^2^)] = 0.047	H-atom parameters constrained
*wR*(*F*^2^) = 0.132	*w* = 1/[σ^2^(*F*_o_^2^) + (0.0753*P*)^2^] where *P* = (*F*_o_^2^ + 2*F*_c_^2^)/3
*S* = 1.05	(Δ/σ)_max_ = 0.001
2664 reflections	Δρ_max_ = 1.52 e Å^−^^3^
159 parameters	Δρ_min_ = −0.78 e Å^−^^3^
0 restraints	Extinction correction: *SHELXL97* (Sheldrick, 2008), Fc^*^=kFc[1+0.001xFc^2^λ^3^/sin(2θ)]^-1/4^
Primary atom site location: structure-invariant direct methods	Extinction coefficient: 0.00176 (18)

### Special details

**Table:**

Geometry. Bond distances, angles etc. have been calculated using the rounded fractional coordinates. All su's are estimated from the variances of the (full) variance-covariance matrix. The cell esds are taken into account in the estimation of distances, angles and torsion angles
Refinement. Refinement of F^2^ against ALL reflections. The weighted R-factor wR and goodness of fit S are based on F^2^, conventional R-factors R are based on F, with F set to zero for negative F^2^. The threshold expression of F^2^ > 2sigma(F^2^) is used only for calculating R-factors(gt) etc. and is not relevant to the choice of reflections for refinement. R-factors based on F^2^ are statistically about twice as large as those based on F, and R- factors based on ALL data will be even larger.

### Fractional atomic coordinates and isotropic or equivalent isotropic displacement parameters (Å^2^)

**Table:**

	*x*	*y*	*z*	*U*_iso_*/*U*_eq_	
Sn1	0.88830 (6)	1.02871 (3)	0.61937 (2)	0.0477 (2)	
S1	0.8813 (3)	0.51496 (13)	0.57463 (11)	0.0572 (6)	
O1	1.0216 (7)	0.7546 (4)	0.6551 (2)	0.0631 (14)	
O2	0.8531 (6)	0.8672 (4)	0.5524 (2)	0.0559 (14)	
O3	0.9257 (8)	1.2230 (4)	0.6901 (3)	0.082 (2)	
N1	0.6645 (8)	0.4207 (4)	0.6350 (3)	0.0534 (18)	
N2	0.5982 (8)	0.6263 (5)	0.5937 (3)	0.0566 (17)	
C1	0.7227 (12)	1.1295 (7)	0.5191 (4)	0.075 (3)	
C2	1.1824 (10)	1.0401 (6)	0.6541 (5)	0.071 (3)	
C3	0.7556 (13)	0.9649 (7)	0.6896 (5)	0.074 (3)	
C4	0.9201 (9)	0.7672 (5)	0.5856 (4)	0.049 (2)	
C5	0.8680 (10)	0.6603 (5)	0.5312 (3)	0.054 (2)	
C6	0.6941 (9)	0.5235 (5)	0.6040 (4)	0.0457 (19)	
C8	0.4562 (10)	0.6241 (8)	0.6160 (4)	0.068 (3)	
C9	0.4131 (11)	0.5250 (8)	0.6490 (5)	0.071 (3)	
C10	0.5225 (10)	0.4220 (6)	0.6579 (4)	0.060 (2)	
C12	0.4868 (13)	0.3083 (7)	0.6930 (5)	0.091 (4)	
H1A	0.61760	1.08170	0.48660	0.1130*	
H1B	0.79770	1.15070	0.49100	0.1130*	
H1C	0.67800	1.20150	0.53440	0.1130*	
H2A	1.23750	1.07840	0.70390	0.1070*	
H2B	1.20970	1.08670	0.61690	0.1070*	
H2C	1.23360	0.96050	0.65730	0.1070*	
H3A	0.72540	0.88110	0.67880	0.1110*	
H3B	0.64240	1.00990	0.67900	0.1110*	
H3C	0.83830	0.97440	0.74320	0.1110*	
H3D	0.96840	1.20950	0.73860	0.1230*	
H3F	0.81920	1.25720	0.67570	0.1230*	
H5A	0.94970	0.66000	0.50340	0.0650*	
H5B	0.74050	0.67200	0.49320	0.0650*	
H8	0.38290	0.69300	0.60890	0.0820*	
H9	0.31430	0.52630	0.66500	0.0850*	
H12A	0.47470	0.24180	0.65860	0.1360*	
H12B	0.37290	0.31660	0.70110	0.1360*	
H12C	0.58980	0.29360	0.74170	0.1360*	

### Atomic displacement parameters (Å^2^)

**Table:**

	*U*^11^	*U*^22^	*U*^33^	*U*^12^	*U*^13^	*U*^23^
Sn1	0.0622 (3)	0.0338 (3)	0.0444 (3)	−0.0027 (2)	0.0186 (2)	0.0027 (2)
S1	0.0757 (11)	0.0341 (7)	0.0735 (11)	−0.0025 (7)	0.0420 (9)	−0.0056 (7)
O1	0.082 (3)	0.050 (2)	0.047 (2)	−0.007 (2)	0.015 (2)	−0.0018 (19)
O2	0.074 (3)	0.038 (2)	0.051 (2)	−0.004 (2)	0.020 (2)	−0.0014 (18)
O3	0.121 (5)	0.046 (3)	0.067 (3)	0.009 (3)	0.024 (3)	−0.009 (2)
N1	0.072 (4)	0.035 (2)	0.054 (3)	−0.010 (2)	0.026 (3)	−0.004 (2)
N2	0.059 (3)	0.048 (3)	0.064 (3)	0.005 (2)	0.026 (3)	0.010 (2)
C1	0.103 (6)	0.057 (4)	0.053 (4)	0.006 (4)	0.018 (4)	0.005 (3)
C2	0.052 (4)	0.056 (4)	0.091 (6)	−0.013 (3)	0.013 (4)	−0.010 (4)
C3	0.098 (6)	0.065 (5)	0.079 (5)	0.005 (4)	0.057 (5)	0.010 (4)
C4	0.063 (4)	0.041 (3)	0.051 (4)	−0.018 (3)	0.030 (3)	−0.011 (3)
C5	0.081 (5)	0.036 (3)	0.053 (3)	−0.012 (3)	0.035 (3)	−0.004 (3)
C6	0.054 (4)	0.034 (3)	0.047 (3)	−0.009 (2)	0.018 (3)	−0.006 (2)
C8	0.054 (4)	0.076 (5)	0.071 (5)	0.014 (4)	0.022 (4)	0.018 (4)
C9	0.053 (4)	0.093 (6)	0.065 (5)	0.001 (4)	0.023 (4)	0.002 (4)
C10	0.069 (4)	0.058 (4)	0.049 (3)	−0.022 (3)	0.021 (3)	−0.003 (3)
C12	0.131 (8)	0.067 (5)	0.088 (6)	−0.033 (5)	0.059 (6)	0.004 (4)

### Geometric parameters (Å, º)

**Table:**

Sn1—O2	2.144 (4)	C10—C12	1.500 (11)
Sn1—O3	2.488 (5)	C1—H1A	0.9600
Sn1—C1	2.127 (7)	C1—H1B	0.9600
Sn1—C2	2.113 (9)	C1—H1C	0.9600
Sn1—C3	2.098 (10)	C2—H2A	0.9600
S1—C5	1.792 (6)	C2—H2B	0.9600
S1—C6	1.752 (8)	C2—H2C	0.9600
O1—C4	1.236 (8)	C3—H3A	0.9600
O2—C4	1.277 (7)	C3—H3B	0.9600
O3—H3D	0.8500	C3—H3C	0.9600
O3—H3F	0.8500	C5—H5A	0.9700
N1—C6	1.342 (8)	C5—H5B	0.9700
N1—C10	1.335 (10)	C8—H8	0.9300
N2—C8	1.328 (10)	C9—H9	0.9300
N2—C6	1.333 (8)	C12—H12A	0.9600
C4—C5	1.512 (8)	C12—H12B	0.9600
C8—C9	1.368 (12)	C12—H12C	0.9600
C9—C10	1.393 (12)		
			
O2—Sn1—O3	176.66 (16)	Sn1—C1—H1C	109.00
O2—Sn1—C1	91.6 (2)	H1A—C1—H1B	109.00
O2—Sn1—C2	95.7 (2)	H1A—C1—H1C	109.00
O2—Sn1—C3	96.3 (2)	H1B—C1—H1C	109.00
O3—Sn1—C1	85.4 (2)	Sn1—C2—H2A	109.00
O3—Sn1—C2	84.4 (2)	Sn1—C2—H2B	109.00
O3—Sn1—C3	86.4 (3)	Sn1—C2—H2C	109.00
C1—Sn1—C2	116.0 (3)	H2A—C2—H2B	109.00
C1—Sn1—C3	117.5 (4)	H2A—C2—H2C	110.00
C2—Sn1—C3	124.6 (3)	H2B—C2—H2C	110.00
C5—S1—C6	101.1 (3)	Sn1—C3—H3A	109.00
Sn1—O2—C4	120.4 (4)	Sn1—C3—H3B	109.00
Sn1—O3—H3F	109.00	Sn1—C3—H3C	110.00
H3D—O3—H3F	109.00	H3A—C3—H3B	109.00
Sn1—O3—H3D	110.00	H3A—C3—H3C	109.00
C6—N1—C10	116.0 (6)	H3B—C3—H3C	109.00
C6—N2—C8	115.1 (6)	S1—C5—H5A	108.00
O1—C4—C5	120.9 (5)	S1—C5—H5B	108.00
O1—C4—O2	125.4 (6)	C4—C5—H5A	108.00
O2—C4—C5	113.8 (5)	C4—C5—H5B	108.00
S1—C5—C4	116.4 (4)	H5A—C5—H5B	107.00
S1—C6—N1	113.5 (5)	N2—C8—H8	119.00
S1—C6—N2	119.1 (5)	C9—C8—H8	119.00
N1—C6—N2	127.4 (7)	C8—C9—H9	121.00
N2—C8—C9	122.9 (8)	C10—C9—H9	121.00
C8—C9—C10	117.7 (8)	C10—C12—H12A	109.00
N1—C10—C9	120.9 (7)	C10—C12—H12B	109.00
N1—C10—C12	117.1 (7)	C10—C12—H12C	109.00
C9—C10—C12	122.1 (8)	H12A—C12—H12B	109.00
Sn1—C1—H1A	109.00	H12A—C12—H12C	109.00
Sn1—C1—H1B	109.00	H12B—C12—H12C	109.00
			
C1—Sn1—O2—C4	−176.2 (6)	C6—N1—C10—C9	0.4 (10)
C2—Sn1—O2—C4	67.5 (6)	C6—N1—C10—C12	−179.8 (6)
C3—Sn1—O2—C4	−58.3 (6)	C8—N2—C6—S1	178.6 (5)
C6—S1—C5—C4	69.3 (6)	C8—N2—C6—N1	−1.1 (10)
C5—S1—C6—N1	176.1 (5)	C6—N2—C8—C9	1.6 (10)
C5—S1—C6—N2	−3.6 (6)	O1—C4—C5—S1	22.7 (10)
Sn1—O2—C4—O1	−6.7 (10)	O2—C4—C5—S1	−158.6 (5)
Sn1—O2—C4—C5	174.7 (5)	N2—C8—C9—C10	−1.2 (12)
C10—N1—C6—S1	−179.6 (5)	C8—C9—C10—N1	0.1 (11)
C10—N1—C6—N2	0.1 (10)	C8—C9—C10—C12	−179.7 (7)

### Hydrogen-bond geometry (Å, º)

**Table:**

*D*—H···*A*	*D*—H	H···*A*	*D*···*A*	*D*—H···*A*
O3—H3*D*···O1^i^	0.85	2.03	2.798 (6)	149
O3—H3*F*···N1^ii^	0.85	2.14	2.884 (7)	146

Symmetry codes: (i) −*x*+2, *y*+1/2, −*z*+3/2; (ii) *x*, *y*+1, *z*.
